# Toll-Like Receptor-3 Is Dispensable for the Innate MicroRNA Response to West Nile Virus (WNV)

**DOI:** 10.1371/journal.pone.0104770

**Published:** 2014-08-15

**Authors:** Pauline E. Chugh, Blossom A. Damania, Dirk P. Dittmer

**Affiliations:** Department of Microbiology and Immunology, Lineberger Comprehensive Cancer Center, Center for AIDS Research, University of North Carolina at Chapel Hill, Chapel Hill, North Carolina, United States of America; French National Center for Scientific Research - Institut de biologie moléculaire et cellulaire, France

## Abstract

The innate immune response to West Nile virus (WNV) infection involves recognition through toll-like receptors (TLRs) and RIG-I-like receptors (RLRs), leading to establishment of an antiviral state. MiRNAs (miRNAs) have been shown to be reliable biomarkers of TLR activation. Here, we sought to evaluate the contribution of TLR3 and miRNAs to the host response to WNV infection. We first analyzed HEK293-NULL and HEK293-TLR3 cells for changes in the innate immune response to infection. The presence of TLR3 did not seem to affect WNV load, infectivity or phosphorylation of IRF3. Analysis of experimentally validated NFκB-responsive genes revealed a WNV-induced signature largely independent of TLR3. Since miRNAs are involved in viral pathogenesis and the innate response to infection, we sought to identify changes in miRNA expression upon infection in the presence or absence of TLR3. MiRNA profiling revealed 70 miRNAs induced following WNV infection in a TLR3-independent manner. Further analysis of predicted gene targets of WNV signature miRNAs revealed genes highly associated with pathways regulating cell death, viral pathogenesis and immune cell trafficking.

## Introduction

West Nile Virus (WNV) is a mosquito-borne neurotropic flavivirus, and closely related to Yellow Fever Virus (YFV) and Dengue virus (DENV). WNV is an enveloped virus and contains a single-stranded, positive-sense RNA genome. The genomic RNA is translated into a single polyprotein, and during viral RNA synthesis dsRNA intermediates are generated in the cytoplasm. WNV is transmitted by mosquitoes and is an emerging pathogen, especially in the Americas, with 5,674 and 2,469 reported cases of WNV in the United States in 2012 and 2013, respectively. [1], [2], [3]. In 2013, 1,494 people were hospitalized with WNV infection with nearly a 10% fatality rate (119/1494) [3]. Cases of WNV transmission by blood transfusion have also been reported [4]. Generally, WNV infection is asymptomatic, although in a small percentage of patients WNV infection can lead to fatal encephalitis, specifically in the elderly, transplant recipients and other immune-compromised hosts including patients infected with HIV. This suggests a pivotal role for the immune response in determining systemic WNV pathogenesis [5].

The innate immune system uses pattern recognition receptors (PRRs) that recognize specific, conserved pathogen-associated molecular patterns (PAMPs)[6]. Several of these PRRs trigger the secretion of type I interferon as part of the innate immune response. The PRRs that have been implicated in triggering the innate response to WNV infection are toll-like receptor (TLR) 3 and 7, Retinoic acid Inducible Gene -I (RIG-I), Melanoma Differentiation Associated protein 5 (MDA5), RIG-I-like receptor 3 (LGP2), and Protein Kinase R (PKR) [7], [8], [9], [10]. These PRRs recognize single-stranded and/or double-stranded RNA, such as the intermediates created by the replicating WNV genome. PRR engagement triggers a signaling cascade leading to the activation of signature transcriptional regulators. Profiling transcriptional events thus provides a means to understand virus infection and innate signaling events. Notably among these transcription factors are NF-κB and IRF-3, which induce inflammatory cytokines, such as type I interferon (IFN-α/β) [11], [12], [13]. The lack of the cytoplasmic helicases MDA5 and RIG-I is associated with a failure to generate an effective immune response to WNV in experimentally infected mice [6], [12], [14], [15], [16], [17]. The common adapter protein for RIG-I and MDA5, MAVS, has been shown to be essential for triggering innate immunity and control of WNV pathogenesis [16], [17]. The absence of PKR signaling or a deficient 2′-5′ oligoadenylate synthase (OAS)/RNaseL pathway also lead to increased susceptibility to WNV infection [18], [19], [20], [21], [22], [23]. TLR7 affects homing of immune cells to the infection site *in vivo* [24]. In sum, multiple and perhaps redundant PRR signaling pathways respond to WNV infection.

TLR3 has a complicated role in the innate response to WNV. In experimentally infected mice, TLR3 can enhance WNV pathogenesis but also protect the host from viral dissemination, CNS inflammation and virulence [25], [26], [27]. TLR3-deficient mice show increased lethal WNV infection and elevated viral burden in the brain compared to wild type mice [25]. TLR3 seems to restrict replication in neurons, leading to decreased neuronal cell death in mice [25]. TLR3 has also been reported to initiate an inflammatory response that leads to breakdown of the blood brain barrier and penetration of WNV, resulting in increased viral load within the brain and increased lethal WNV infection [27].

Less is known about TLR3's cell autonomous role in WNV infection. Studies of WNV in culture have focused on the role of individual WNV proteins, such as the nonstructural protein NS1. Expression of NS1 was shown to inhibit TLR3-induced interferon and NFκB activation and to abrogate the TLR3-mediated antiviral response in HeLa and HEK293 cells [28], [29]. Others reported that transient or stable expression of NS1 proteins from WNV and two related flaviviruses failed to inhibit TLR3 signaling in HeLa and HEK293 cells [30].

To further our understanding of the role of TLR3 in WNV infection, we turned to microRNA (miRNA) profiling as a novel tool to characterize the host response. MiRNAs are a class of 21 to 25-nt non-coding RNAs that are conserved across species, including humans and insects. They modulate a wide range of cellular processes such as cell cycle control, replication, apoptosis and immunity [31], [32]. MiRNA expression can also be altered upon stimulation of TLR signaling. Activation of TLR3 by polyI:C treatment induces miR-29b, miR-29c, miR-148b and miR-152 in tumor cell lines [33]. TLR3 has also been shown to upregulate expression of miR-155, miR-146 and miR-147 in monocytes and macrophages [34], [35]. Some viruses employ virally-encoded miRNAs to mediate establishment of a persistent or latent viral infection. Although flaviviruses themselves are not known to encode miRNAs in mammalian cells [36], [37], KUN-miR-1, a small RNA derived from the WNV 3′ UTR, has been discovered in infected mosquito cells and affects viral replication [38]. Slonchak et al. also found that in *Aedes albopictus* cells, WNV infection downregulates mosquito-specific aae-miR-2940 to restrict viral replication [39]. In mammalian cells, it is likely that the host cell utilizes miRNAs that can affect its ability to fight off viral infection, either indirectly by targeting key innate immune signaling molecules or by directly binding to the viral genome. Conversely, the WNV may generally suppress or specifically modulate the cellular miRNA profile to benefit viral replication and spread [40], [41], [42], [43]. Virus-derived small RNAs may also play a functional role in the response to WNV infection [44]. Smith et al. found that cellular miRNA hs_154 was induced by WNV infection and contributed to virus-mediated cell death in HEK293 and SK-N-MC cells [37]. Therefore, a comprehensive analysis of miRNA expression following WNV infection may reveal additional miRNAs important for viral pathogenesis and provide insight into the regulation of the miRNA response to WNV.

In this study, we determined the effect of WNV infection on the cellular miRNA repertoire using quantitative real-time PCR assays (TaqMan) directed against ∼450 well validated human miRNAs. Since TLR3 signaling plays an important but complicated role in WNV infection, we analyzed the miRNA profile in the presence and absence of TLR3. The cellular response to infection (pIRF3, NFκB) and the WNV-induced miRNA signature were virtually indistinguishable in two otherwise isogenic cell lines, as was WNV replication. This suggests that TLR3 signaling is dispensable for the miRNA response to WNV infection.

## Materials and Methods

### Cells and viruses

For consistency we chose the same cell type, HEK293, as in prior studies. HEK293-Null and HEK293-TLR3 cells were from Invivogen, Inc (catalog # 293-Null and 293-htlr3). They were maintained in DMEM supplemented with 10% FBS, Pen/Strep and Blasticidin (10 µg/ml). WNV strain NY99 was obtained from the ATCC. Virus was titered by plaque assay on Vero cells or BHK cells as described [45].

### Virus infection

HEK293-Null and -TLR3-expressing cells were seeded at 1×10^7^ cells in a 10 cm dish and infected with 1×10^8^ PFU WNV (MOI = 10). As a negative control, equivalent amounts of virus were inactivated using the methylene blue light inactivation protocol [45], [46] or by formalin inactivation (0.05% in phosphate buffered saline) for 24 hours followed by three washes in phosphate buffered saline (PBS). As a positive control, cells were exposed to the TLR3 agonist polyI:C (5 µg/ml added to the cell supernatant, Sigma catalog #42424).

### Western blotting

Cells (1×10^6^ cells/well) were either left uninfected, treated with inactivated (in.) virus or infected with West Nile Virus (MOI = 10). Cells were harvested at various time points post-infection and lysed in NP40 lysis buffer (50 mM Tris, 150 mM NaCl, 1% NP40 supplemented with protease inhibitor (Sigma catalog #P8340), 0.03% β-mercaptoethanol, 50 mM NaF, 1 mM sodium vanadate, 30 mM β-glycerophosphate and 1 mM PMSF). 20 µg of protein (as determined by Pierce BCA Protein Assay) was loaded per well and run on a 12% SDS - polyacrylamide gel. Following transfer of proteins to a nitrocellulose membrane (HyBond, Amersham Biosciences, Inc.), blots were incubated with rabbit anti-phospho-IRF3 antibody (Epitomics, Inc.) at a 1∶1000 dilution and mouse anti-beta actin at 1∶3000 dilution overnight at 4°C and then washed three times with Tris-buffered saline with Tween (TBST). Immunoblots were then probed with anti-rabbit and anti-mouse HRP-conjugated antibodies at 1∶5000 dilution (Vector Laboratories, Inc.) for 2 hours at room temperature and washed again three times with TBST. An ECL substrate kit (Pierce Inc. #32209) with “Blue Devil” X-ray film (Genesee#30–100) was used for detection. Exposures from 10 seconds to 1 minute were taken, showing similar results.

### Immunofluorescence

HEK293-Null and HEK293-TLR3 cells were plated on coverslips at 5×10^5^ cells prior to infection with WNV at an MOI = 10. Cells were fixed in a 3% paraformaldehyde/PBS solution for 15 minutes at room temperature followed by incubation overnight in a blocking solution of 10% goat serum (Vector Labs) in phosphate buffered saline (PBS) with 0.2% Triton X-100. 100 µl of mouse monoclonal anti-WNV E (1∶20 dilution; Virostat Inc.) was spotted onto each coverslip and incubated overnight at 4°C. For detection of phosphorylated p65, P-NF-kappaB p65 (S536) primary antibody (clone 93H1, 1∶100 dilution; Cell Signaling) was used. Following three washes with PBS-0.2% Triton with 2% BSA, anti-mouse Texas Red secondary antibody (Vector Labs Inc. #TI-2000) and/or anti-rabbit Fluorescein secondary antibody (Vector Labs Inc. #FI-1000) was added to the coverslips at 1∶500 dilution for 2 hours. Cells were then washed three times with PBS-0.2% Triton X-100 with 2% BSA and stained with 0.2 µg/ml DAPI (Sigma Inc.) prior to mounting in VectaShield (Vector Labs Inc.). Images were taken on a Leica DM4000B microscope with a Q-Imaging Retiga-2000RV camera and HCX-PL-APO 506187 lens at 630× magnification. De-convoluted, merged images are shown (Simple PCI 6 software, 10 iterations RB or RGB fields).

### WNV titration on the xCelligence system

Approximately 5×10^3^ cells/well were plated on an E-plate 16 (Acea Biosciences). After 4 hours, cells were infected with serial dilutions of West Nile Virus (MOI = 5, 0.5, 0.05, etc.). Cell index as a measure of viability was monitored continuously over a period of 100 hours. The time of peak cell index (in hours), which reflects the onset of WNV-induced cytopathic effects, was also plotted for each cell type against MOI for graphical representation of data.

### RNA isolation and cDNA synthesis

Total RNA was isolated using Trizol (Sigma Inc.) as previously described [47]. RNA quality was determined using the Agilent RNA Nano 6000 kit as per the manufacturer's protocol (Agilent Inc.). All samples had a RNA integrity number (RIN) of >8.0. To obtain cDNA, equal amounts of RNA as determined by Nanodrop quantification were DNase treated (Ambion Inc.). The High Capacity cDNA synthesis kit (Applied Biosystems Inc.) was then used to reverse transcribe 100 ng DNase-treated RNA sample as per the manufacturer's instructions.

### TLR3 RT-PCR

The cDNA was isolated from HEK293-Null and HEK293-TLR3 cell lines as above. This was used as input for PCR of TLR3 expression using primers obtained from SA Biosciences (catalog #PPH01803E). PCR products were then run on a 1% agarose gel and visualized on a BioRad Gel Doc.

### NFκB array

The cDNA isolated from the same WNV-infected Null and TLR3 samples was also used as input for the 96-well qPCR array of NFκB-responsive genes [48]. The Tecan Freedom Evo liquid handling robot was used to setup the NFκB array for each sample in duplicate. 10 µl reactions were run using SYBR Green-based technology and the Roche LC480 real-time PCR system. ddCT values were determined and data was clustered as described in the calculations section.

### Taqman miRNA profiling

The Taqman miRNA reverse transcription kit was used to individually reverse transcribe 448 human miRNAs (Applied Biosystems Inc.). Reactions were performed in 96-well format using 10 ng total RNA per reaction. All reactions were assembled using a Tecan FreedomEvo robot to limit pipetting error [49]. QPCR reactions were setup using the complete array of mature miRNA specific primers and Taqman Universal PCR Master Mix in a 384-well format. Each 11 µl reaction used 5 µl 2×PCR master mix, 2 µl 10×QPCR primer (1.8× final primer concentration) and 4 µl cDNA. QPCR was carried out in quadruplicate on a LightCycler480 (Roche Inc.) using the standard Taqman cycling program for 55 cycles.

### Calculations and statistical analysis of miRNA profiling data

To gain a general overview of the data set, we first calculated the median raw expression measurement (CT) for each of the miRNAs (n = 448). MiRNAs and structural small RNAs (RNU6B, etc) were measured using the same calculation methods, beginning with the raw CT readout from the Taqman small RNA assay. MiRNAs were then normalized to the trimmed mean of 12 different structural RNAs. In Figure S1, panel A, the cumulative density for n = 448 miRNAs measurements across 40 samples is shown, summarizing 14,240 individual qPCR reactions. The maximum cycle number was 55 as can be seen by a peak at median CT of 55. Approximately 30% of all miRNAs had a median CT of 55, i.e. these were not detected in a meaningful fraction of the samples. MiRNAs with a CT>50 across all conditions (i.e. not expressed) were excluded from further analysis, yielding a total of 356 miRNAs. Next, we calculated the median absolute deviation (mad) for each miRNA across all samples. The mad is a more robust measure of variability compared to the standard deviation. In Figure S1, panel B, the mad CT is plotted against the median CT for each miRNA. The cumulative density of the mads was also plotted (Figure S1, panel C). Approximately 17% of miRNAs had a mad  = 0, which indicates that they did not change across any of the experimental conditions. This indicates miRNAs that were not detected in any of the samples. As seen in Figure S1 panel B, miRNAs with a median CT>45 show a strong linear relationship with the mad (blue line). This suggests systematic, rather than biological variation. Hence, we set the maximum CT = 45 cycles for all subsequent analyses. After this adjustment, the median CTs followed a normal distribution, which allowed us to use the sensitive t-test for individual comparisons.

The CT values were scaled by sample and subjected to unsupervised hierarchical clustering using “Ward's” agglomeration method and the “Manhattan” distance metric. This method was used to generate the heatmap representation of the data. As an alternative approach, we used a second unsupervised genetic clustering approach based on a Gaussian distribution model (data not shown). This yielded essentially similar results, but also suggested additional miRNAs as TLR3-dependent, such as miR-542-3p, which exhibited some degree of differential expression.

To improve upon the sensitivity of our analysis even further, we pursued specific comparisons. By-and-large, specific hypothesis testing is a more sensitive analytical approach than unsupervised clustering, which works best if there exist easily discernable groups or clusters of genes. To detect changes between HEK293-NULL and HEK293-TLR3, we conducted a t-test using the time points 2, 4, and 8 hrs post treatment. This yielded uncorrected p-values representing the likelihood that any one miRNA differed in expression between two treatments. These raw p values were then adjusted for multiple comparisons testing using the q-value method by Storey and Tibshirani [50]. Upon q-value correction for polyI:C treatment and WNV infection, respectively (pi0 = 0.907; 0.861), we obtained miRNAs with q≤0.05.

### WNV viral load

Viral load was determined by RT-qPCR as per our published procedures [51] with primers: WNV Env F: 5′-TCAGCGATCTCTCCACCAAAG-3′; WNV Env R: 5′-GGGTCAGCACGTTTGTCATTG-3′. The following oligo was diluted to generate a standard curve to determine copy number: WNV Env Oligo: TCAGCGATCTCTCCACCAAAG CTGCGTGCCCGA*CCATGG*GAGAAGCTCACAATGACAAACGTGCTGACCC . A novel, engineered Nco I restriction site is denoted in italics within the Env oligo sequence. Cutting the amplification products with NcoI thus allowed us to exclude contamination by the standard. Reactions were run on a Roche LightCycler 480 and absolute copy number was determined by comparison to plaque assay.

### Gene ontology analysis

WNV-induced miRNAs obtained from Taqman profiling were used for further analysis of functional downstream targets. The dataset of miRBase IDs was input to Ingenuity Pathway Analysis software (www.ingenuity.com) and predicted targets were obtained [52]. The mRNA targets were then filtered through several different parameters, excluding non-human genes and including only experimentally validated targets. The top functional roles of the remaining 550 targets were determined using the Ingenuity software, and the statistical significance (p value) and number of genes involved in each functional category are shown. For the miRNA targets of individual Heatmap cluster subsets, Ingenuity analysis was performed similarly. In cases where the number of experimentally validated targets for the entire cluster was below 15, predicted mRNA targets with high confidence were also included in the functional analysis.

## Results

### Assessing the role of TLR3 in the cell autonomous response to WNV infection

**Figure 1:**
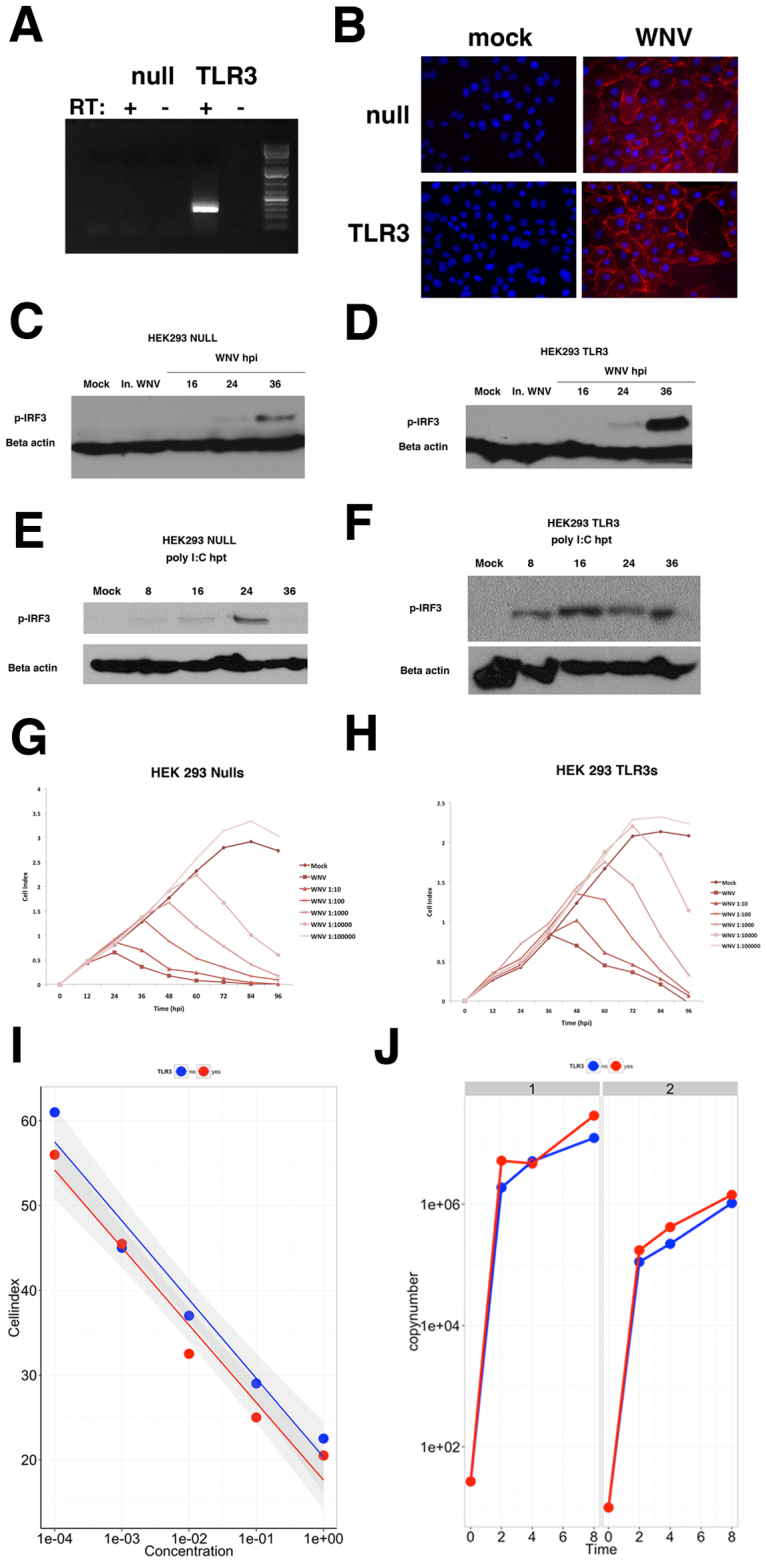
The cellular response to WNV infection is unaffected by TLR3 status. (A) RT-PCR analysis of TLR3 expression in HEK293-Null and HEK293-TLR3 cells. RT negative reactions were performed as a control. (B) Immunofluorescence images of cells either mock- or WNV-infected are shown. Cells were stained with WNV E antibody (red) and DAPI (blue) and merged, deconvoluted images are shown at 630× magnification. (C–F) Western blots of phosphorylated IRF3 and beta actin in HEK293-Null and HEK293-TLR3 cells at various time points following WNV infection or post-treatment with the TLR3 agonist poly I:C. Mock infected cells and cells treated with inactivated WNV are shown as controls. (G, H) xCelligence analysis of cell viability following infection with serial dilutions of WNV NY99 (MOI = 5 to 0.00005). WNV-induced cytopathogenicity curves are shown for HEK293-Null (G) and HEK293-TLR3 cells (H). Reads were taken every 30 minutes for a period of 100 hours. (I) WNV-induced CPE kinetics were obtained by plotting the time of peak cell index for each sample against the concentration of WNV. HEK293-Nulls are represented by the blue line while HEK293-TLR3s are denoted by the red line. This panel shows a linear regression across 5 log dilutions. Shown are the individual cell indices at each dilution, the calculated robust linear regression lines (R^2^ = 0.968) and in the gray shading the standard error (SE) band across the entire dilution range. (J) Viral load was determined by QPCR analysis for WNV envelope protein using a set of known WNV envelope standards. Panel J shows WNV copy number as determined by real-time qPCR using two biological replicate experiments (1 and 2) conducted one week apart. There was no significant growth difference.

Since previous studies of the role of TLR3 in WNV infection have revealed both a protective host response as well as enhanced *in vivo* pathogenesis, we characterized the cell autonomous response to infection in the presence or absence of TLR3. HEK293-TLR cells are widely used to dissect single TLR-specific signaling events [53], [54], [55]. They have also been the cell line of choice in prior studies on the role of individual WNV proteins, such as NS1. HEK293-TLR3 cells ectopically express only TLR3 and HEK293-Null cells have very little to no expression of any of the known TLRs. Minimal residual activity of TLR3 has been reported in HEK293-Nulls (www.invivogen.com). Therefore, we first confirmed the TLR3 status by RT-PCR. Only the HEK293-TLR3 cells expressed TLR3 while TLR3 message levels were not detected in the HEK293-Null cells, suggesting a ∼1,000-fold lower level in these cells (Figure 1A).

HEK293-Null and HEK293-TLR3 cells were infected with WNV at an MOI of 5 and at 24 hours post-infection evaluated for the presence of WNV envelope protein (E) by immunofluorescence. We chose this MOI and time course to ensure that every cell is infected and represents synchronous WNV infection in culture. Indeed, WNV E staining showed that 100% of HEK 293 Null were permissive to WNV infection at 24 hours (Figure 1B). No differences in WNV infectivity were observed in HEK293-TLR3 cells compared to HEK293-Nulls (Figure 1B). This finding is consistent with prior studies [25], [56].

We next investigated IRF3 activation. Upon infection, WNV delays IRF3 phosphorylation and nuclear translocation to allow for viral replication prior to the activation of anti-inflammatory genes [12], [57], [58]. Figure 1C, D shows that IRF3 phosphorylation is unaffected by inactivated virus and is triggered by WNV infection between 24 and 36 hours post-infection. Similar kinetics of IRF3 phosphorylation were observed regardless of the presence or absence of TLR3; however, the amounts of phosphorylated IRF3 appeared slightly elevated in TLR3-expressing cells compared to Nulls at late times post infection. This is likely due to the convergence of TLR3-dependent and –independent pathways of IRF3 activation in HEK293-TLR3 cells at late times post-infection (Figure 1D, 36 hpi).

We next assessed the IRF3 response to the TLR3 agonist polyI:C. Treatment of HEK293-TLR3 cells with poly I:C led to sustained phosphorylation of IRF3 as early as 8 hours post-treatment, confirming that ectopically expressed TLR3 is functional. By comparison to polyI:C treatment, IRF3 phosphorylation was substantially delayed in HEK293-TLR3 cells following WNV infection (Figure 1D, F). We also functionally tested the HEK293-NULL cells for their response to the TLR3 agonist polyI:C (10 µg/ml) added to the supernatant. Hyperstimulation of HEK293-NULLs with poly I:C resulted in a modest, delayed phosphorylation of IRF3 at one late time point, i.e. 24 hours post-stimulation (Figure 1E). This is in contrast to HEK293-TLR3 cells, where robust IRF3 phosphorylation was evident by 8 hours and sustained for up to 36 hours. This demonstrates that the TLR3 response is severely curtailed in HEK293-Null cells. It is not clear whether the minimal signal at very late times was the result of polyI:C recognition by residual TLR3 or secondary events at late times. Figure 1C-F serves as a control to show that the timing of our infection process recapitulates the known biology of TLR3 and WNV infection at the time when we profiled samples and the cells (and hence RNAs) retain viability at a high MOI, i.e. 0 up to 8 or at most 16 hours (not 36 hours). During the 0-16 hour time frame, IRF3 experiences maximal phosphorylation in response to pI:C when TLR3 is expressed (panel E and F), as has been documented since the discovery of TLR3. In the 0–16 hour time frame, IRF3 is not phosphorylated by WNV infection regardless of TLR3 status, as has been documented many times [29], [57], [58], [59].

The antiviral, innate immune response to WNV triggered by PRR signaling eventually leads to cell death of infected cells. Therefore, we next compared WNV cytopathogenicity in HEK293-Null and HEK293-TLR3 cells using the xCelligence method for determining cell death [60]. This novel assay provides detailed insight into the kinetics of viral cytopathic effects. The xCelligence platform is capable of continuously monitoring cellular parameters such as adhesion, cell viability, cytotoxicity and cell number using a label-free method. Cells are grown on specialized plates coated with a microelectrode that measures impedance, recorded as “Cell Index”. Using this method, we continuously monitored the cytopathic effects (CPE) of viral infection in relation to MOI and also determined the onset of virus-induced CPE for a given sample. HEK293-Null and HEK293-TLR3 cells were plated at 5000 cells/well and infected with 10-fold serial dilutions of WNV NY99 (MOI of 5, 0.5, 0.05…to 0.00005). Cytopathic effects induced by viral infection were assessed continuously over a period of 100 hours. The differences in the cell index (y-axis) reflect differences in cell adhesion between HEK293- NULL and HEK293-TLR3 cells, the latter being much less adherent. Importantly, both HEK293-Null and –TLR3 cells were equally susceptible to WNV-induced CPE (Figure 1G and H).

The peak cell index for each sample provides us insight into the kinetics of WNV-induced cytotoxicity. We determined the WNV-induced CPE kinetics for the different viral dilutions in the presence and absence of TLR3. HEK293-TLR3 cells that were mock-infected seemed to have slightly increased cell viability, however this did not affect CPE kinetics. Figure 1I shows that the kinetics of WNV-induced CPE follows a linear trend and correlates well with the multiplicity of infection for each serial dilution (R^2^ = 0.968). The onset of WNV-induced CPE was independent of TLR3 status as determined by ANOVA comparison of the MOI dependence fit curves (Figure 1G–I).

Since there were no differences in infectivity or cytopathic effects regardless of TLR3 status, we next assessed whether TLR3 had any effect on WNV replication. Intracellular WNV replication was determined by RT-qPCR using WNV oligonucleotides in serial dilution to generate a standard curve for copy number. WNV genomes were readily detected as early as 2 hours post-infection and levels of intracellular virus increased steadily throughout the course of infection (Figure 1J). Cellular TLR3 status had no significant effect on WNV intracellular RNA replication by ANOVA using time, replicate and TLR3 status as independent factors. This suggests that TLR3 does not limit intracellular virus genome replication. Taken together, analysis of the characteristic cellular responses to infection revealed that although high levels of TLR3 expression increased IRF3 phosphorylation in response to WNV infection at late times, the TLR3 status did not affect infectivity, viral genome replication or WNV-induced CPE.

### NFκB signaling in response to WNV

**Figure 2:**
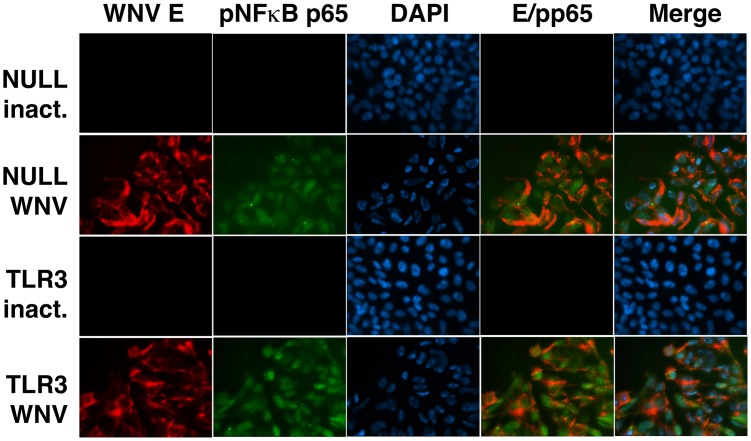
WNV infection induces phosphorylated NFκB p65. HEK293-NULL and HEK293-TLR3 cells were infected with WNV (MOI = 10) and fixed in 3% paraformaldehyde at 6 hours post-infection. Methylene blue inactivated virus was used as a control. Immunofluorescence assay (IFA) was performed for phosphorylated p65 (green), WNV E (red) and DAPI (blue). Images are shown at 630× as individual panels, WNV E/p65 merge and full merged images of WNV E/p65/DAPI.

Activation and nuclear translocation of NFκB and the subsequent transcription of NFκB-responsive genes is a common marker for triggering the host antiviral response. To test the NFκB response to WNV, we performed an immunofluorescence assay to monitor the status of phosphorylated NFκB p65 following infection with WNV. Figure 2 demonstrates that upon WNV infection, p65 becomes phosphorylated and translocates to the nucleus in both infected HEK293-NULL and HEK293-TLR3 cells. This event happens early during infection, as phosphorylated p65 was detected at 6 hours post-infection (Figure 2). WNV E protein was detected following infection of both HEK293-NULL and HEK293-TLR3 cells, while inactivated virus failed to yield detectable phosphorylated p65 or WNV E staining. This demonstrates that in contrast to IRF3 phosphorylation, which is delayed until 36 hours following WNV infection, NFκB signaling can be activated at early times post-infection and with it NFκB responsive mRNA and miRNA transcription. NFκB signaling represents another aspect of the antiviral response that can be profiled to characterize the initial cellular response to WNV infection.

**Figure 3:**
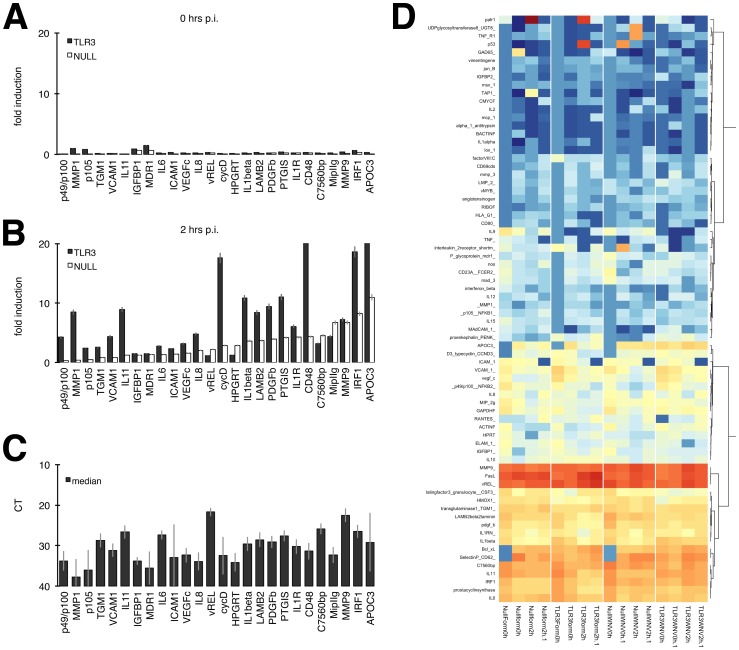
WNV infection induces a unique NFκB-responsive gene expression profile. HEK293-Null and –TLR3 cells under the specified conditions were analyzed for gene expression profiles using a qPCR array consisting of ∼90 NFκB-responsive genes. (A,B) Fold induction of genes was calculated by comparing WNV-infected samples to cells treated with formalin-inactivated virus. WNV-induced gene expression is shown at 0 hours post-infection (A) and 2 hours post-infection (B) with WNV (MOI = 10). Black bars denote TLR3-expressing HEK293 cells while open bars represent HEK293-Nulls. (C) The median CT across all samples was calculated and is shown +/− SEM for each gene analyzed. All genes shown were abundantly expressed (CT<35). (D) Heatmap of NFκB genes profiled. Highly abundant miRNAs are indicated by red and non-expressed miRNAs by blue color. Yellow indicates an intermediate expression level. This is also shown underneath the heatmap. The miRNAs were clustered based on expression.

We previously developed a qPCR-based array of ∼90 NFκB-responsive, experimentally validated genes [48]. To test the hypothesis that TLR3 signaling influenced the quality of the NFκB response, we analyzed the gene expression profile of NFκB target genes in response to WNV infection. Prior to WNV infection, both HEK293-Null and HEK293-TLR3 cells had low basal levels of NFκB-responsive gene transcription (Figure 3A, 0 hpi). Induction of NFκB-responsive genes in WNV-infected cells was compared to gene expression in cells treated with formalin-inactivated virus, allowing for a signature of genes induced specifically by productive WNV infection. Changes in gene expression were detected as early as 2 hours post-infection, suggesting that NFκB activation occurs much earlier than IRF3 activation, which occurred at about 36 hours post-infection. HEK293-Nulls had a WNV-induced NFκB-responsive gene expression profile with moderate induction of genes observed at early times post-infection (Figure 3B). By comparison, HEK293-TLR3s displayed significantly higher induction of key NFκB-responsive genes. The WNV-induced NFκB-responsive gene signature included IL-6, IL-8, IL-1β signaling, IRF1, subunits of NFκB and matrix metalloproteinases (MMPs), which are known to be involved in WNV pathogenesis [61], [62], [63], [64], [65], [66], [67], [68]. Figure 3C shows the median CT across all samples and demonstrates that all mRNAs within this WNV-induced NFκB signature were present in both cell types at significant levels (i.e. within the linear range of the qPCR assay) and Figure 3D shows a heatmap of all NFkB-responsive genes profiled. Many well-known NFκB target genes are not expressed in HEK293 cells at sufficiently high levels to allow for meaningful statistical analysis (even though heatmap-based clustering by the all inclusive nature of this algorithm will assign an output color). We therefore limited our statistical analysis to those mRNAs which were robustly detectable in all biological and technical replicates, with a median CT cutoff of 38 cycles across n = 18 samples (see Figure 3C). The majority of those were induced to higher levels in WNV-infected TLR3 cells compared to WNV-infected Null cells. This was, however, a quantitative difference, not a qualitative difference. We could not identify a single NFκB responsive mRNA, which was induced only in WNV-infected TLR3 cells, but not in WNV-infected Null cells; and we could not identify a single NFκB responsive mRNA, which was induced only in WNV-infected Null cells, but not in WNV-infected TLR3 cells. These data suggest that NFκB is engaged in both HEK293-Null cells as well as in HEK293-TLR3 cells. Whereas many NFκB targets were common to both cells, indicative of the shared RIG-I/TRAF/TBK signaling axis, others such as MMP1, cyclin D, IL-1 beta and CD48 were more dramatically induced in TLR3 positive cells compared to HEK293-NULLS.

### miRNA profile of WNV infection in TLR3 positive and TLR3 Null cells

Since miRNAs play a role in the host response to viral infection and TLR signaling, we sought to identify the miRNAs that change in response to WNV infection. This would provide us with another class of biomolecules to characterize TLR3 dependent aspects of the innate immune response to WNV infection. We employed a Taqman-based QPCR array to quantify levels of 448 known mature human miRNAs. Cells were infected with WNV at an MOI of 5 or exposed to an equal amount of inactivated virus. The miRNA profiles were determined at 0, 2, 4 and 8 hours post-infection, and relative levels of expression were determined.

**Figure 4:**
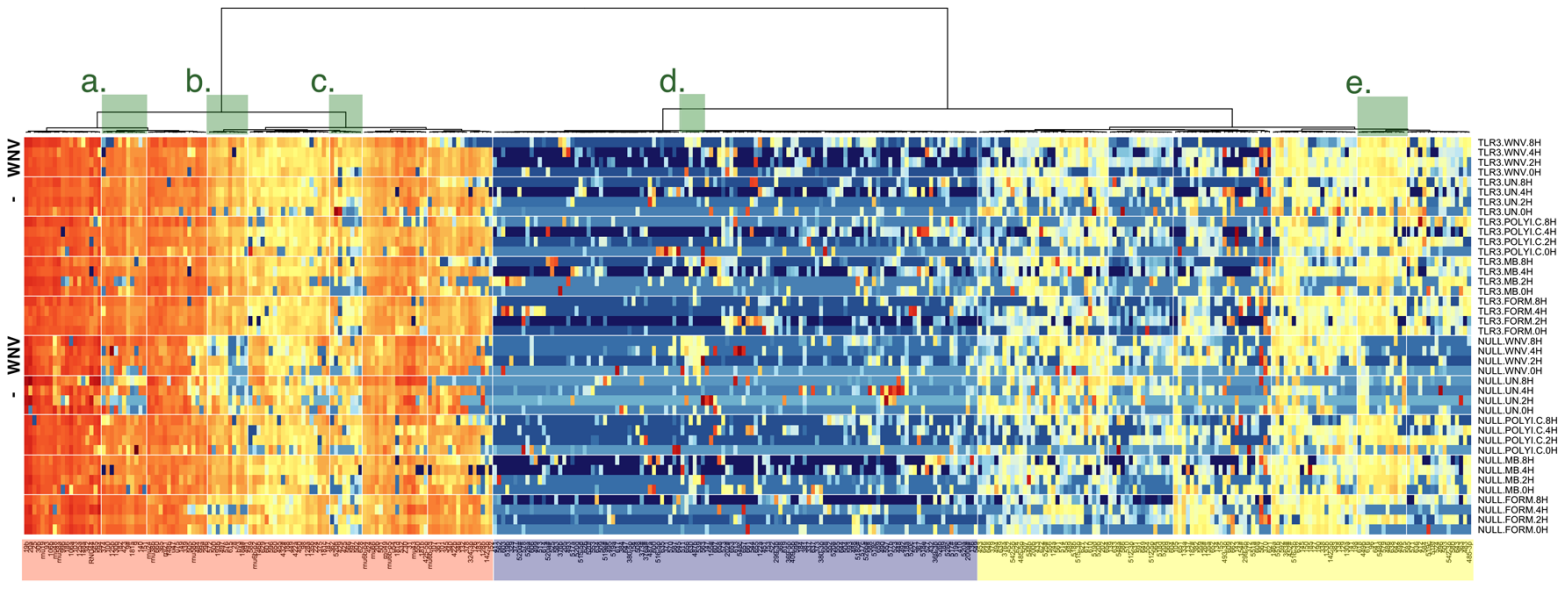
MiRNA profiling reveals a WNV-induced signature. Large-scale miRNA profiling was carried out in HEK293-Null and –TLR3 cells using Taqman-based qPCR. Heatmap representation of all raw data points for the experiment. Sample abbreviations are as follows: UN – mock infected cells; WNV – WNV-infected cells; polyIC – cells treated with TLR3 agonist polyI:C; MB – cells exposed to methylene blue-inactivated WNV; Form – cells exposed to formalin-inactivated WNV. Highly abundant miRNAs are indicated by red and non-expressed miRNAs by blue color. Yellow indicates an intermediate expression level. This is also shown underneath the heatmap. The miRNAs were clustered based on expression. The dendrogram is shown on the top and interesting clusters are highlighted in green and indicated by small letters (a high resolution figure is presented as Figure S2).

The CT values were scaled by sample and unsupervised clustering was performed as described in the methods. The data from these algorithms and QC analysis of the profiling data are shown in Figure S1 and raw profiling data is shown in Table S9. In Figure 4, orange/red indicates miRNAs that are more highly expressed, blue indicates miRNAs that are absent, and yellow indicates miRNAs expressed at intermediate levels. A large number of miRNAs were not detectable in any sample, as indicated by the blue bar underlay. The exception within this group is cluster “d”. Cluster “d” comprised miRNAs, which were upregulated only in HEK293-NULL cells after WNV infection. These included: miR-511, miR-563, miR-656, miR-630, miR-487b and miR-539. Cluster “a” comprised miRNAs which were abundantly expressed in all samples except untreated HEK293-NULL cells and contained the miRNAs: miR-151, miR-140, miR-9, miR-9*, miR-181a, miR-374, miR-100, miR-125b, miR-30b, miR-182 and miR-423. Cluster “b” comprised miRNAs which were consistently expressed in HEK293-TLR3 and decreased in mock and WNV-infected HEK293-NULL cells and contained the miRNAs: miR-592, miR-500, miR-193b, miR-497, miR-576, miR-615, miR-28, miR-18a* and miR-491. Cluster “c” contained miRNAs that were induced by continued culture (i.e. increased with time cells spent in culture). These were: miR-362, miR-520b, miR-429, miR-425, miR-566, miR-597, miR-502 and miR-628. Finally, cluster “e” contained miRNAs, which were detectable in HEK293-TLR3 cells, but were decreased in uninfected and WNV-infected HEK293-NULL cells. These included miR-618, miR-414d, miR-616, miR-96, miR-641, miR-548d, miR-579, miR-486, miR-638, miR-642, miR-498 and miR-575.

To further improve upon the sensitivity of our analysis, we selected a subset of miRNAs, requiring that in at least one sample the miRNA was present in significant amounts (CT≤28) and normalized based on the 10% trimmed mean across 12 abundantly expressed structural RNAs (rnu6b-p3, rnu19, rnu24, rnu38b, rnu43, rnu44, rnu48, rnu58a, rnu58b, rpl21, u47, u75). This yielded the conventional dCT values as the basis of our analysis for a set of 124 miRNAs.

**Figure 5:**
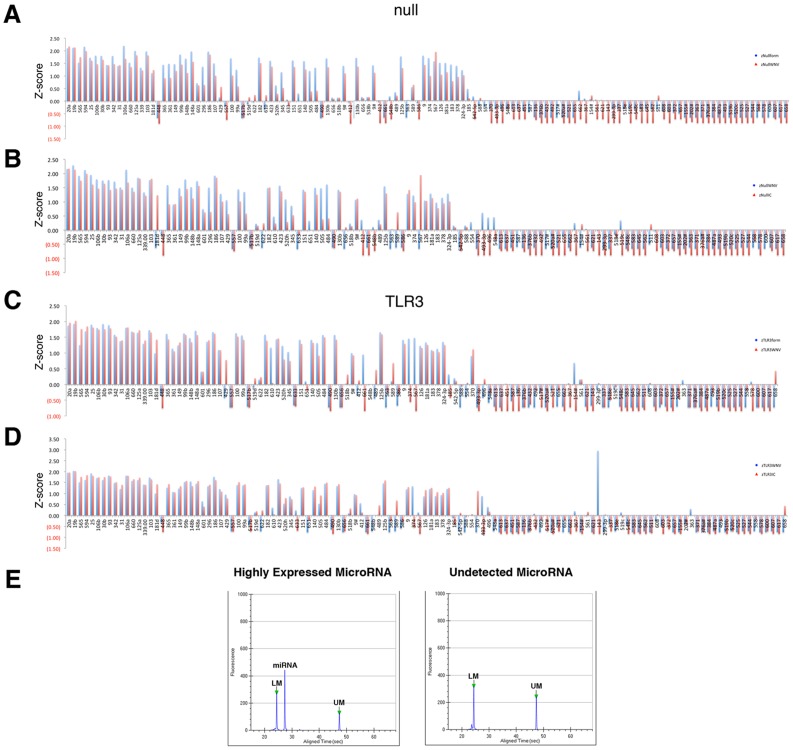
Directed Comparison of abundant miRNAs. All miRNAs with at least one data point had a CT≤28 were selected, normalized to stable small RNAs, and the median CT across the 2, 4, and 8 hr time point was calculated. These were further standardized to yield a Z score, which reflects relative changes. The Z score is shown on the vertical axis and miRNAs on the horizontal axis. The Z scores were ordered by the level of uninfected HEK293-Null (A, B) or HEK293-TLR3 (C, D) cells. Panels (A) and (C) compare the response of WNV to formalin-inactivated virus. Panels (B) and (D) compare the response of WNV infection to polyI:C stimulation. (E) Caliper LabChip analysis of a highly expressed and undetected miRNA. qPCR products of miRNA assays were run on a Caliper LabChip GX and the resulting electropheregram is shown. The sample yields a single peak corresponding to the expressed miRNA and two assay marker bands (UM – upper marker and LM – lower marker). The undetected miRNA electropheregram shows only an upper and lower marker peak with very little to no background signal, confirming the high specificity of this assay.

To identify miRNAs that changed consistently upon infection, we calculated for each the median across the 2, 4, and 8-hour time-point and Z-standardized those by sample. As a result we could calculate relative expression ratios between any two conditions. The majority of miRNAs did not change drastically upon treatment, particularly in HEK293-TLR3 cells (Figure 5). There was evidence of generally lower miRNA expression upon any treatment in HEK293-NULL cells, which could be evidence of a general delay in proliferation or miRNA biosynthesis. Overall, WNV and formalin-inactivated WNV induced similar trends in miRNA changes. This would be consistent with the experience of formalin-inactivated WNV without adjuvant in the vaccine setting, where either particle induces a similar adaptive immune response [69].

The most interesting comparison was in the HEK293 TLR3 cells (Figure 5C,D). Here, the background variation compared to uninfected cells was so low for most miRNAs that changes in individual miRNAs upon treatment stood out. Formalin-inactivated WNV induced the miRNAs miR-567, miR-661, miR-154*, miR-578 and miR-610, whereas live WNV infection did not (Figure 5, panel C). Both preparations repressed a number of miRNAs (miR-662, miR-600, miR-544, miR-487a, miR-493-3p, miR-562). Treatment with the TLR3 agonist poly:IC dramatically induced mir-143, whereas formalin-treated or live WNV did not. Other miRNAs in this class were miR-367, miR-517*, miR-567 and miR-493-3p. Note that we only included miRNAs in the comparison where at least one of the data points had a CT<28, i.e. be so abundant that we could confirm detection by Caliper nanofluidics based gel analysis (panel E), which yields both a gel image and electropheregram for each lane. We have recently used Caliper gel analysis as a method of visualizing miRNA expression [70]. By separating the qPCR products on the Caliper nanofluidics system which has higher resolution than agarose gels, we are able to detect that miRNAs that are highly expressed produce a single species, whereas undetected miRNAs and primers (CT>40) do not produce a band of appropriate size (Figure 5E). This is observed through the visualization of one single, specific peak at the expected size of miRNAs and confirms the high specificity of the miRNA assays.

To detect changes between HEK293-NULL and HEK293-TLR3, we conducted a t-test using the time points 2, 4, and 8 hrs post treatment. This yielded uncorrected p-values representing the likelihood that any one miRNA differed in expression between two treatments. Comparing changes in miRNA levels upon poly I:C stimulation of either NULL or TLR3-expressing HEK293 cells, 7 miRNAs remained significantly changed to <0.05 after adjustment for multiple comparisons using the q-value method [50]. These were as follows: miR-20a, miR-99a, miR-517* (miR-517b was significant in raw p-value), miR-519b, miR-583, miR-651 and miR-662. Comparing changes in miRNA levels upon WNV stimulation of either NULL or TLR3-expressing HEK293 cells, our analysis yielded 45/125 (36%) miRNAs with an unadjusted p≤0.05. Few miRNAs remained significantly changed to <0.05 after adjustment for multiple comparisons. These were as follows: miR-20a, miR-99a, miR-517, miR-683, miR-587, miR-651 and miR-662.

Overall, the statistical analyses of our profiling data demonstrate that there were very few changes in miRNA expression following WNV infection or treatment with inactivated virus. Changes in the miRNA profile induced by WNV were rather consistent between cell types and the WNV miRNA signature was not significantly affected by the presence or absence of TLR3. These data further suggest that the majority of WNV-regulated miRNAs at early times post-infection (8 hrs) are not TLR3-dependent, but may depend on signaling through other PRRs such as RIG-I and MDA5 and/or other sensors like PKR (RIG-I, MDA5 and PKR are fully functional in HEK293 cells) [71], [72], [73], [74], [75], [76], [77], [78], [79].

**Figure 6:**
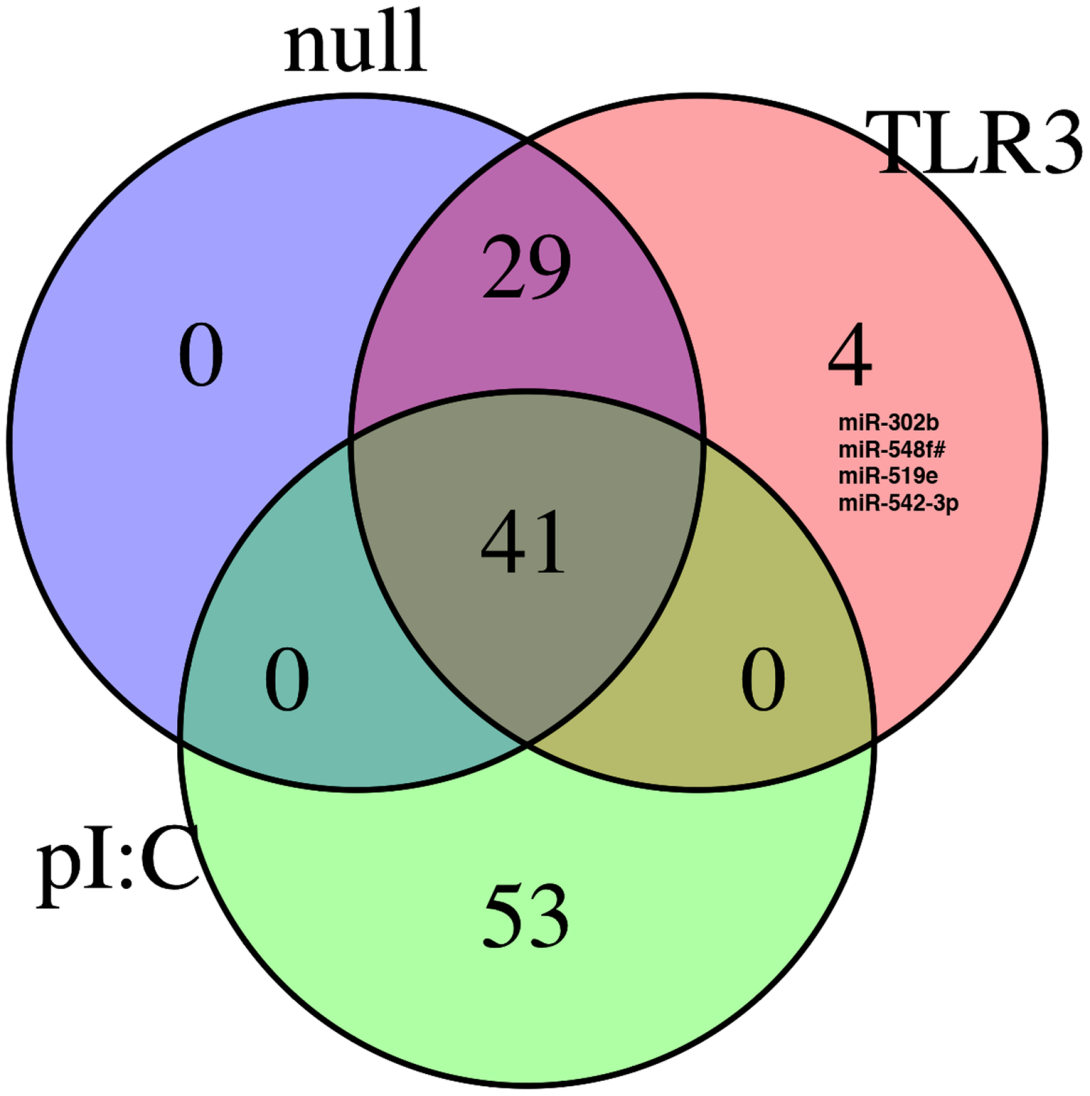
Venn diagram of miRNAs that were induced in HEK293 Null and HEK293 TLR3 cells. Venn diagram representation of miRNAs induced following WNV infection of HEK293-Null and HEK293-TLR3 cells or polyI:C stimulation of HEK293-TLR3 cells. The number of shared, induced miRNAs are shown for each sample subset. A detailed list of the miRNAs induced for each category represented in the Venn diagram (HEK293-NULL +WNV, HEK293-TLR3 + WNV and HEK293-TLR3 + polyI:C treatment) is shown in Table S6.

We then compared the miRNA response to WNV infection and stimulation of TLR3 via polyI:C treatment. Figure 6 shows a Venn diagram illustrating that the majority of WNV dependent miRNAs (n = 70) were induced in both HEK293-TLR3 and –NULL cells. Only 4 were induced only in HEK293-TLR3 cells. Furthermore, the miRNA profile following engagement of TLR3 signaling through polyI:C treatment yielded a relatively distinct profile compared with WNV infection: 41 miRNAs were shared between WNV infected cells and polyI:C induced TLR cells but none were specific to HEK293-TLR3 cells. There were 53 miRNAs uniquely induced just by polyI:C in HEK293-TLR3 cells. These data suggest that WNV-induced miRNAs predominantly respond to signaling events independent of TLR3 or that miRNA response to WNV was substantially dampened.

### WNV-induced miRNA targets have functional roles in infection

Since miRNAs often work in combination to confer a functional phenotype, we next investigated the combined effect of miRNA changes in WNV-infected cells by analyzing induced miRNAs from the WNV signature for predicted functional roles in WNV pathogenesis. WNV-specific miRNAs were analyzed using Ingenuity Pathway Analysis (IPA). Experimentally validated miRNA targets were obtained and subjected to gene ontology analysis, separating the target genes into functional categories. We also performed pathway analysis of predicted miRNA targets in each unique cluster from Figure 4 (Tables S1,S2,S3,S4,S5 for clusters a-e, respectively). MiRNAs induced by treatment with the TLR3 agonist were also analyzed by pathway analysis of predicted targets (Tables S7, S8).

**Figure 7:**
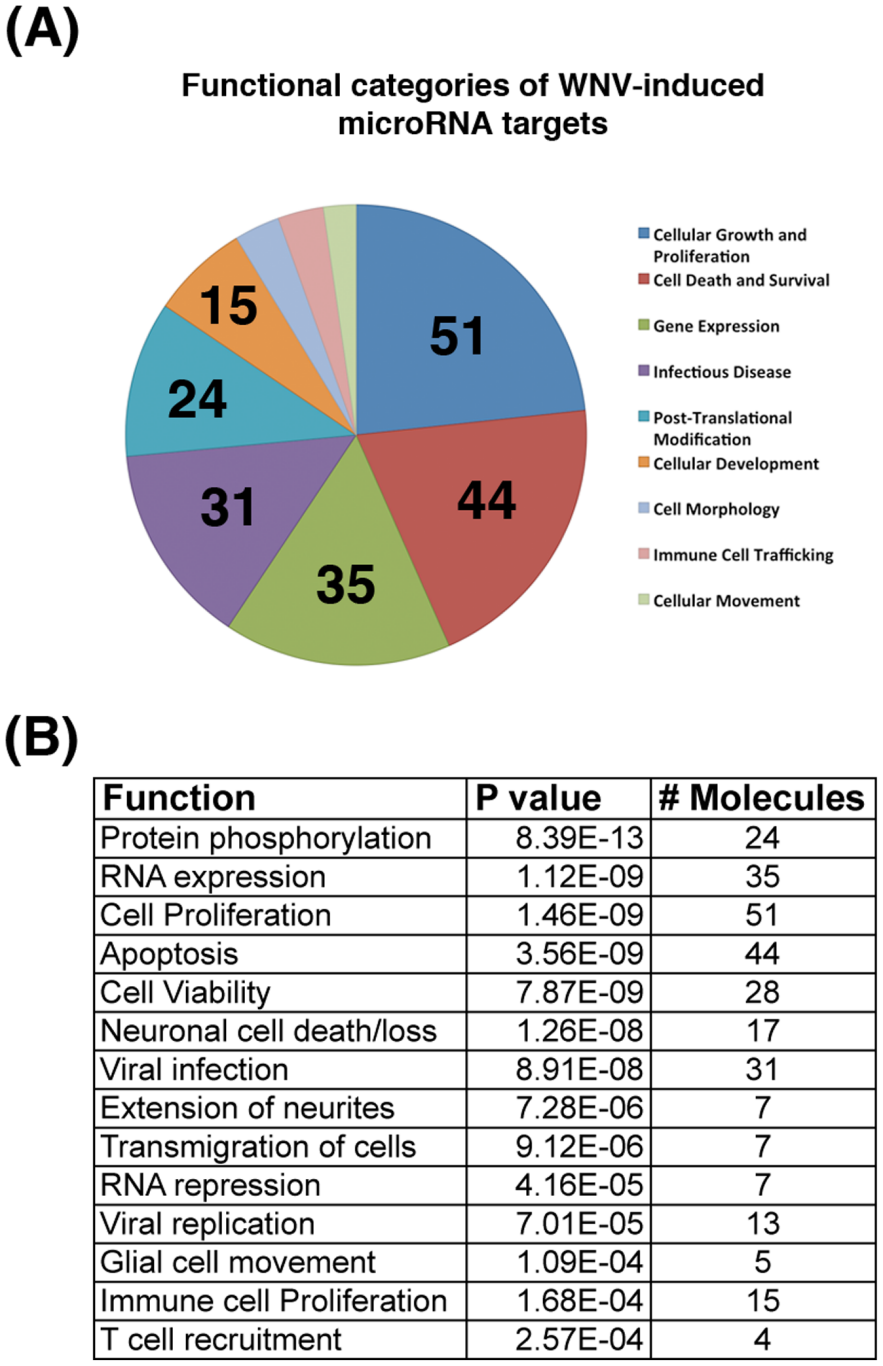
WNV-induced miRNAs target processes functionally relevant to WNV pathogenesis. WNV signature miRNAs were analyzed for functions of predicted targets using Ingenuity Pathway Analysis software (IPA). Predicted targets of these miRNAs were determined and filtered through several criteria to yield functional categories for target genes. (A) The top functional categories are represented and the number of miRNA target genes in each category is denoted. (B) More detailed functions of the WNV-induced miRNA targets. Functions are shown along with statistical significance (P value) and the number of genes that correspond to each function. The p value was calculated in IPA using the right-tailed Fisher Exact Test and compares the number of focus genes that participate in a given functional process and the total number of genes known to be associated with the function in the selected set of miRNA target genes.

Several functional categories were significantly linked to the WNV-induced miRNA signature (Figure 7). The largest number of target genes affected functions such as cell proliferation, cell death, gene expression and infectious disease (Figure 7A). WNV is known to affect cell death and proliferation throughout the course of infection to allow for efficient viral replication and spread of the virus through apoptosis and cell lysis. The infectious disease category also included many WNV-linked functions, such as immune cell trafficking and viral replication. These are further specified in the more detailed functions of target genes shown in Figure 7B. Several processes related to proliferation, recruitment and transmigration of cells were also listed, suggesting a potential role of these miRNA targets in WNV neuropathogenesis. Functional analysis remains to be performed and will further shed light on the role of these miRNAs and specific targets in WNV pathogenesis. As a control, we also analyzed the target genes of miRNAs induced by infection of B cells with an unrelated virus, Kaposi sarcoma Herpesvirus (KSHV) [80]. This verified that the WNV-induced miRNA targets had a distinct gene ontology profile from this control. There were several pathways that were significantly changed upon infection with WNV but considered N/A or not significant in KSHV-infected cells (data not shown). Notably, these included the immune and neural related pathways.

## Discussion

The host response to WNV infection is regulated by multiple PRRs that sense the invading viral ss and dsRNA: RIG-I, TLR3 and PKR. RLR signaling involving RIG-I, MDA5 and the common adapter protein IPS-1 is essential to control WNV infection in mice and in culture [12], [16], [17], [81]. TLR3 has been shown to influence WNV pathogenesis both positively and negatively in vivo, by aiding in mounting a protective immune response and by enhancing infection and neuropathogenesis [25], [26], [27], [29].

Most of the studies so far used *in vivo* infection models. To further characterize the cell-intrinsic role of TLR3, we assessed viral replication and the cellular response to WNV in the presence or absence of functional TLR3 (Figure 1). Minimal residual activity of TLR3 has been reported in HEK293-Nulls (www.invivogen.com). However, TLR3 message levels were undetectable in HEK293-NULLs and hyperstimulation of these cells with polyI:C resulted in a significantly delayed IRF3 response compared to HEK293-TLR3 cells, which may in fact be due to secondary unspecific events. Therefore, HEK293-NULLs were considered to have very low levels of TLR3 activity and our profiling was conducted at early times (<8 hours). TLR3-overexpressing HEK293 and HEK293NULL cells could be productively infected with WNV (Figure 1), yielding similar intracellular viral loads and CPE profiles in response to infection. WNV staining by immunofluorescence was also identical regardless of cellular TLR3 status. Only HEK293-TLR3 responded to polyI:C treatment by phosphorylation of IRF3 at 8 hours. We observed increased phosphorylation of IRF3 in HEK293-TLR3 cells compared to HEK293-NULLs likely due to the convergence of TLR3-dependent and RIG-I-dependent signaling events in response to WNV at late times post-infection. This characteristic, delayed activation of IRF3, was observed in HEK293-TLR3 and HEK293-NULL upon WNV infection. It was independent of TLR3 status, as was early activation of nuclear phospho-NFκB (Figure 2). This would indicate that NFκB signaling was engaged by WNV independent of TLR3.

More in-depth analysis of downstream NFκB-responsive genes demonstrated that WNV does indeed induce a specific NFκB-regulated signature (Figure 3). This unique profile consists of many cytokines and inflammatory genes involved in mounting an effective immune response against WNV infection. Though HEK293-NULLs mounted a robust NFκB signature following infection, the majority of NFκB targets were upregulated to a greater extent in HEK293-TLR3 cells following WNV infection. This quantitative enhancement is presumably due to the additive effect of NFκB activation via both TLR3-dependent and TLR3-independent mechanisms. In addition, we observed qualitative differences in the NFκB response profile (Figure 3).

MiRNAs control many cellular processes including apoptosis, oncogenesis and the regulation of the immune responses [40], [82]. We profiled host cellular miRNAs in response to WNV infection (Figures 4, 5). The majority of miRNAs did not change, demonstrating that there was not a global impact on mature miRNA levels within the timeframe that is relevant for productive infection. One of the possible reasons why miRNAs were not affected is their high stability. Since mature miRNAs have a relatively long half-life of ∼5 days [83], the overall levels of mature miRNAs may change very little during infection with rapidly replicating viruses. Alternatively, miRNA biosynthesis may be globally downregulated. Shapiro et al. demonstrated that upon infection with an RNA virus, Drosha is dramatically relocalized to the cytoplasm [84]. Others also reported global dampening of miRNA expression in productive viral infections, perhaps in an effort to enhance the immediate interferon response [41], [42]. In addition, differentiated cells show inhibition of the RISC compared to pluripotent cells and may have attenuated activity of another component of the miRNA processing machinery, Dicer (reviewed in [40]).

Although there was no global miRNA effect induced by WNV, we identified and validated specific miRNAs that constitute a WNV–specific miRNA signature. Most of these miRNAs were induced in TLR3-positive as well as TLR-negative cells, suggesting that they respond to TLR-independent signaling events (Figure 6). This was in contrast to poly I:C responsive miRNAs. The cytoplasmic helicases RIG-I and MDA5 play an important role in WNV recognition and the host cell response to infection [12], [17], [57], [81], [85], [86], [87]. Specifically, a model was proposed that RIG-I may be responsible for the immediate early response to infection and that the function of TLR3 may be in sustaining this response [17], [57], [58], [81], [85], [86]. PKR and the OAS/RNaseL pathway also remain important dsRNA sensors during WNV infection [19], [20], [21], [22], [88]. Such a model would be consistent with our profiling data, as we observed dramatic RNA changes already at early times (2–4 hours) after infection, whereas TLR3-dependent phosphorylation of IRF3 was not induced until much later (Figure 1C, 36 hpi).

WNV was previously shown to inhibit TLR3 signaling through NS1 although another study of the nonstructural proteins from multiple flaviviruses failed to see this phenotype [28], [29], [30]. We observed a different miRNA profile for polyI:C stimulated TLR3 cells compared to WNV infected TLR3 cells, as demonstrated in the Venn diagram of induced miRNAs (Figure 7). These miRNAs are also shown in detail in Table S6. This, together with delayed IRF3 phosphorylation (Figure 1), confirms that WNV indeed abrogates TLR3 signaling.

Smith et al. also reported a miRNA profiling study in response to WNV infection in HEK293 and SK-N-MC cells and identified a subset of about 24 miRNAs induced in WNV-infected cells [37]. It is difficult to compare these studies directly due to differences in cells, experimental methods and profiling techniques (qPCR versus microarray). Differences in profiling techniques alone are known to contribute to the variation of datasets [70]. Smith et al. reported that a molecule termed hs_154 was significantly induced following WNV infection [37]. At the time of their publication and design of this TaqMan array, this was not a known miRNA in miRbase. It has since been added to mirBase and is known as hsa-miR-6124 (GGGAAAAGGAAGGGGGAGGA). Out of the 24 miRNAs Smith et al. reported as WNV-induced, 6 miRNAs were included in our Taqman miRNA profiling assays. We confirmed 4 of these (miR-33, miR-299-5p, miR-335, miR-563), which were also induced in our study. All had very low expression levels, with miR-563 being the most consistent among them. One of the 6 miRNAs was downregulated rather than induced (miR-494) and one (miR-603) had an inconclusive profile in the Taqman assay. We were not able to demonstrate a functional impact of any one miRNA on WNV replication in this system (data not shown). This is perhaps not unexpected because WNV in tissue culture is extremely robust, much more so than during primary infection of specialized immune cells such as Langerhans or endothelial cells. In culture, WNV and most other “rapid” viruses are not affected by any host miRNAs [89].

## Conclusions

Taken together, our data reveal a WNV associated miRNA signature that includes miRNAs that target functionally relevant processes important to WNV pathogenesis. Comparison of Null and TLR3-expressing HEK293 cells further demonstrates that other PRRs primarily initiate the NFκB-driven and the miRNA response to WNV infection and that TLR3 expression does not directly affect viral replication or cytotoxicity. Collectively, this suggests that while TLR3 may play an important role in viral entry to the CNS and BBB disruption, TLR-independent mechanisms such as RIG-I and MDA5/IPS-1 primarily control the immediate, cellular response to infection, including characteristic, WNV-induced miRNA responses.

## Supporting Information

Figure S1 **QC analysis of miRNA profiling data and application of statistical algorithms.** Large-scale miRNA profiling was carried out in HEK293-Null and –TLR3 cells using Taqman-based qPCR. Methods used to generate this analysis are described in detail within the Methods section. (A) shows the cumulative distribution (on the vertical axis) of the median CT (on the horizontal axis) for each of the miRNA assays across all samples. (B) shows the median absolute deviation (mad) on the vertical axis compared to the median CT on the horizontal axis for each of the miRNA assays across all samples (Using median and mad is more outlier resistant than using mean and standard deviation). (C) shows the cumulative distribution (on the vertical axis) of mad CT (on the horizontal axis) for each of the miRNA assays across all samples.
(TIF)

Figure S2 **High Resolution Image of WNV-induced miRNA profiling figure (** **Figure 4** **).** Large-scale miRNA profiling was carried out in HEK293-Null and –TLR3 cells using Taqman-based qPCR. Heatmap representation of all raw data points for the experiment. Highly abundant miRNAs are indicated by red and non-expressed miRNAs by blue color. Yellow indicates an intermediate expression level. This is also shown underneath the heatmap. The miRNAs were clustered based on expression. The dendrogram is shown on the top and interesting clusters are highlighted in green and indicated by small letters.
(PNG)

Table S1 **Ingenuity Functional Analysis of miRNA Targets from Heatmap Cluster “a.”**
(DOCX)

Table S2 **Ingenuity Functional Analysis of miRNA Targets from Heatmap Cluster “b.”**
(DOCX)

Table S3 **Ingenuity Functional Analysis of miRNA Targets from Heatmap Cluster “c.”**
(DOCX)

Table S4 **Ingenuity Functional Analysis of miRNA Targets from Heatmap Cluster “d.”**
(DOCX)

Table S5 **Ingenuity Functional Analysis of miRNA Targets from Heatmap Cluster “e.”**
(DOCX)

Table S6 **MicroRNAs from Venn Diagram analysis.** This table shows a list of microRNAs induced by WNV infection and polyI:C treatment in HEK293 cells. Each column details the microRNAs for the Venn diagram analysis (Figure 7). MicroRNAs unique to each treatment column are denoted in bold font with grey background. MicroRNAs that met the hard criteria defined for analysis in Figure 5 are denoted with a dash (−).
(DOCX)

Table S7 **Ingenuity Functional Analysis of miRNA Targets from polyI:C induced microRNAs (Unique to pI:C treatment).** MicroRNAs uniquely upregulated by polyI:C treatment in HEK293-TLR3 cells were analyzed using Ingenuity pathway analysis software. Gene ontology categories and functions of these microRNA targets are shown.
(DOCX)

Table S8 **Ingenuity Functional Analysis of miRNA Targets from abundantly expressed polyI:C induced microRNAs (Unique to pI:C treatment).** Abundantly expressed microRNAs that were uniquely upregulated following polyI:C treatment in HEK293-TLR3 cells were identified using the hard cutoff criteria described for Figure 5. Targets of these microRNAs were analyzed using Ingenuity pathway analysis software and gene ontology categories and functions are shown.
(DOCX)

Table S9 **CTs of miRNA Profiling Data.** Table of the raw CTs from all samples profiled.
(XLS)
