# Supplementary figures and images for: Toll-Like Receptor-3 Is Dispensable for the Innate MicroRNA Response to West Nile Virus (WNV)
Source: PLoS One. 2014 Aug 15;9(8):e104770. doi: 10.1371/journal.pone.0104770 (PMC4134228; doi:10.1371/journal.pone.0104770)

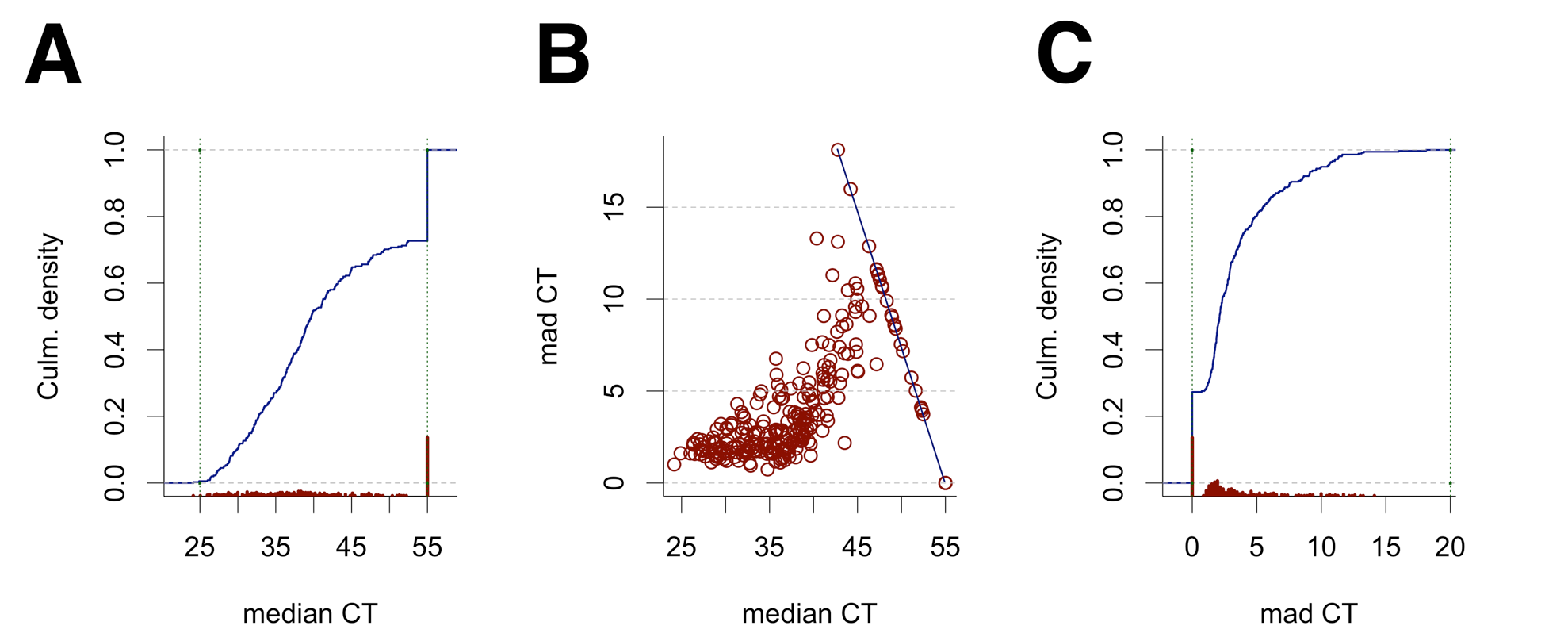

Supplement: Figure S1 — QC analysis of miRNA profiling data and application of statistical algorithms. Large-scale miRNA profiling was carried out in HEK293-Null and –TLR3 cells using Taqman-based qPCR. Methods used to generate this analysis are described in detail within the Methods section. (A) shows the cumulative distribution (on the vertical axis) of the median CT (on the horizontal axis) for each of the miRNA assays across all samples. (B) shows the median absolute deviation (mad) on the vertical axis compared to the median CT on the horizontal axis for each of the miRNA assays across all samples (Using median and mad is more outlier resistant than using mean and standard deviation). (C) shows the cumulative distribution (on the vertical axis) of mad CT (on the horizontal axis) for each of the miRNA assays across all samples. (TIF) [file pone.0104770.s001.tif]

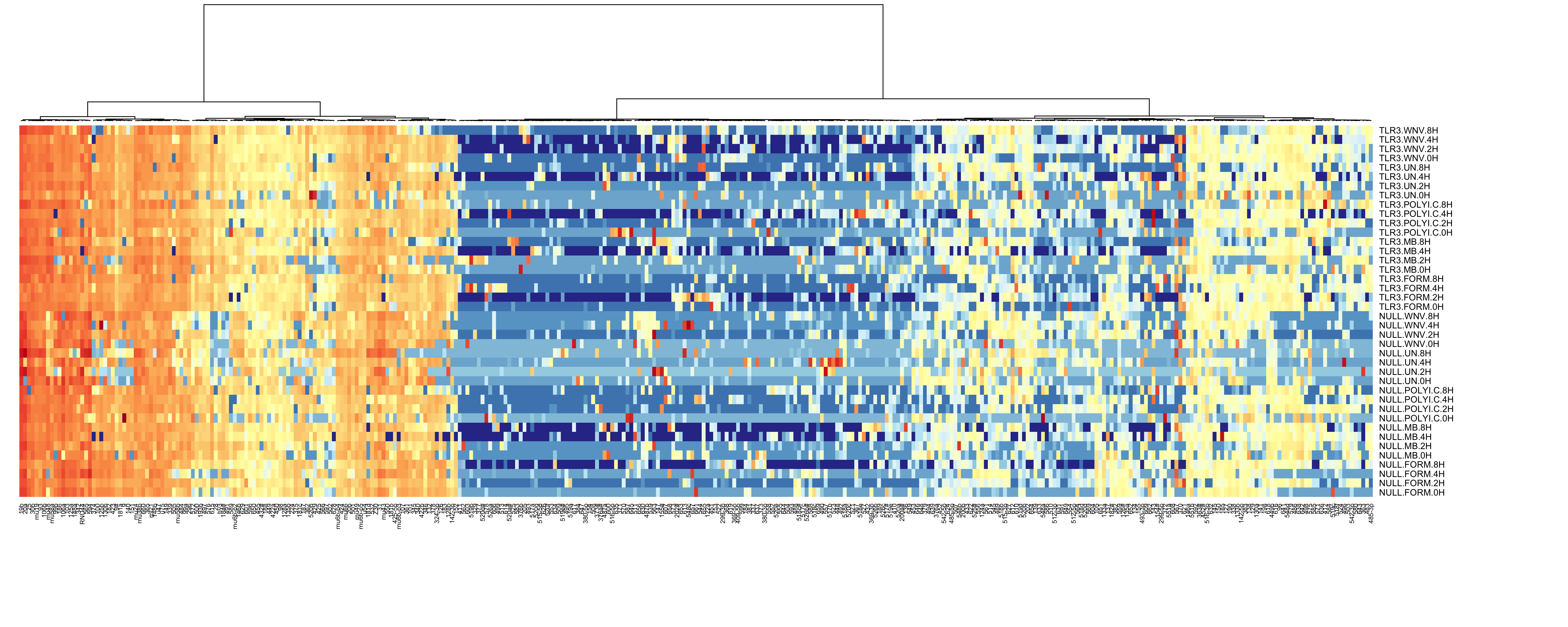

Supplement: Figure S2 — High Resolution Image of WNV-induced miRNA profiling figure ( Figure 4 ). Large-scale miRNA profiling was carried out in HEK293-Null and –TLR3 cells using Taqman-based qPCR. Heatmap representation of all raw data points for the experiment. Highly abundant miRNAs are indicated by red and non-expressed miRNAs by blue color. Yellow indicates an intermediate expression level. This is also shown underneath the heatmap. The miRNAs were clustered based on expression. The dendrogram is shown on the top and interesting clusters are highlighted in green and indicated by small letters. (PNG) [file pone.0104770.s002.png]
