# Supplementary material for: Toll-Like Receptor-3 Is Dispensable for the Innate MicroRNA Response to West Nile Virus (WNV)
Source: PLoS One. 2014 Aug 15;9(8):e104770. doi: 10.1371/journal.pone.0104770 (PMC4134228; doi:10.1371/journal.pone.0104770)
Supplement: Table S1 — Ingenuity Functional Analysis of miRNA Targets from Heatmap Cluster “a.” (DOCX) [file pone.0104770.s003.docx]

**Table S1.**

**Ingenuity Functional Analysis of miRNA Targets from Heatmap Cluster “a.”**

| **GO Category** | **Function** | **p-Value** | **# Molecules** |
| --- | --- | --- | --- |
| Cellular Development | Differentiation | 1.33E-26 | 49 |
| Cell Death and Survival | Apoptosis | 2.38E-24 | 57 |
| Cancer | Tumorigenesis | 1.90E-21 | 30 |
| Cell Morphology | Morphology | 1.00E-19 | 36 |
| Cellular Growth and Proliferation | Proliferation | 2.02E-19 | 57 |
| Cellular Movement | Migration | 3.75E-19 | 42 |
| Gene Expression | Transcription | 4.04E-18 | 38 |
| Cell Cycle | G1/S phase transition | 6.85E-15 | 15 |
| Cellular Function and Maintenance | Homeostasis | 9.29E-14 | 33 |
| Cellular Growth and Proliferation | Colony formation | 5.38E-12 | 18 |
