# Supplementary material for: Toll-Like Receptor-3 Is Dispensable for the Innate MicroRNA Response to West Nile Virus (WNV)
Source: PLoS One. 2014 Aug 15;9(8):e104770. doi: 10.1371/journal.pone.0104770 (PMC4134228; doi:10.1371/journal.pone.0104770)
Supplement: Table S2 — Ingenuity Functional Analysis of miRNA Targets from Heatmap Cluster “b.” (DOCX) [file pone.0104770.s004.docx]

**Table S2.**

**Ingenuity Functional Analysis of miRNA targets from Heatmap Cluster “b”.**

| **GO Category** | **Function** | **p-Value** | **# Molecules** |
| --- | --- | --- | --- |
| Cell Cycle | Progression | 4.56E-18 | 40 |
| Cancer | Cell transformation | 1.55E-15 | 25 |
| Cell Death and Survival | Cell death | 3.16E-13 | 24 |
| Cellular Movement | Invasion | 7.69E-13 | 29 |
| Cellular Development | Proliferation | 9.42E-13 | 26 |
| Gene Expression | RNA transactivation | 1.63E-09 | 21 |
| DNA Replication | DNA synthesis | 2.33E-09 | 20 |
| Cell Cycle | Senescence | 2.68E-09 | 13 |
| Cancer | Tumorigenesis | 9.99E-09 | 10 |
| Cellular Movement | Homing | 1.25E-08 | 20 |
