# Supplementary material for: Toll-Like Receptor-3 Is Dispensable for the Innate MicroRNA Response to West Nile Virus (WNV)
Source: PLoS One. 2014 Aug 15;9(8):e104770. doi: 10.1371/journal.pone.0104770 (PMC4134228; doi:10.1371/journal.pone.0104770)
Supplement: Table S3 — Ingenuity Functional Analysis of miRNA Targets from Heatmap Cluster “c.” (DOCX) [file pone.0104770.s005.docx]

**Table S3.**

**Ingenuity Functional Analysis of miRNA Targets from Heatmap Cluster “c.”**

| **GO Category** | **Function** | **p-Value** | **# Molecules** |
| --- | --- | --- | --- |
| Cellular Movement | Invasion | 1.22E-09 | 21 |
| Cell Cycle | Cell cycle progression | 1.79E-09 | 24 |
| Cell Cycle | Senescence | 9.25E-09 | 11 |
| Cancer | Transformation | 1.19E-08 | 15 |
| Cell Death and Survival | Cell viability | 1.79E-08 | 10 |
| Cell Morphology | Morphology | 2.77E-08 | 25 |
| Cellular Development | Differentiation | 3.25E-08 | 31 |
| Gene Expression | RNA Transcription | 5.44E-08 | 28 |
| Gene Expression | RNA Repression | 1.69E-06 | 8 |
| Cellular Organization | Cytoskeletal Formation | 1.76E-06 | 11 |
