# Supplementary material for: Toll-Like Receptor-3 Is Dispensable for the Innate MicroRNA Response to West Nile Virus (WNV)
Source: PLoS One. 2014 Aug 15;9(8):e104770. doi: 10.1371/journal.pone.0104770 (PMC4134228; doi:10.1371/journal.pone.0104770)
Supplement: Table S4 — Ingenuity Functional Analysis of miRNA Targets from Heatmap Cluster “d.” (DOCX) [file pone.0104770.s006.docx]

**Table S4.**

**Ingenuity Functional Analysis of miRNA Targets from Heatmap Cluster “d.”**

| **GO Category** | **Function** | **p-Value** | **# Molecules** |
| --- | --- | --- | --- |
| Cancer | Cancer | 6.98E-28 | 427 |
| Gene Expression | Transcription | 3.50E-16 | 113 |
| Gene Expression | RNA Expression | 2.35E-12 | 149 |
| Cellular Assembly | Microtubule dynamics | 1.26E-11 | 92 |
| Cellular Assembly | Cytoskeletal organization | 1.36E-11 | 102 |
| Cell Morphology | Cellular Protrusions | 5.40E-11 | 70 |
| Post-Translational Modification | Protein phosphorylation | 8.68E-09 | 61 |
| Gene Expression | Transactivation | 2.79E-08 | 56 |
| Cell Morphology | Morphology | 4.44E-08 | 99 |
| Protein Synthesis | Oligomerization | 1.54E-07 | 23 |
