# Supplementary material for: Toll-Like Receptor-3 Is Dispensable for the Innate MicroRNA Response to West Nile Virus (WNV)
Source: PLoS One. 2014 Aug 15;9(8):e104770. doi: 10.1371/journal.pone.0104770 (PMC4134228; doi:10.1371/journal.pone.0104770)
Supplement: Table S5 — Ingenuity Functional Analysis of miRNA Targets from Heatmap Cluster “e.” (DOCX) [file pone.0104770.s007.docx]

**Table S5.**

**Ingenuity Functional Analysis of miRNA Targets from Heatmap Cluster “e.”**

| **GO Category** | **Function** | **p-Value** | **# Molecules** |
| --- | --- | --- | --- |
| Cancer | Cancer | 1.56E-28 | 478 |
| Cell Morphology | Morphology | 5.89E-24 | 153 |
| Gene Expression | Transcription | 1.54E-17 | 165 |
| Cellular Development | Differentiation | 4.31E-14 | 172 |
| Gene Expression | DNA expression | 7.30E-14 | 122 |
| Cellular Assembly | Cytoskeletal organization | 1.16E-13 | 118 |
| Post-Translational Modification | Protein phosphorylation | 2.21E-13 | 78 |
| Cell Morphology | Neuron Morphology | 2.54E-13 | 41 |
| Cellular Maintenance | Microtubule dynamics | 9.37E-13 | 104 |
| Cell Morphology | Cellular Protrusions | 3.22E-11 | 77 |
| Cellular Assembly | Neurite Growth | 5.13E-10 | 57 |
