# Supplementary material for: Toll-Like Receptor-3 Is Dispensable for the Innate MicroRNA Response to West Nile Virus (WNV)
Source: PLoS One. 2014 Aug 15;9(8):e104770. doi: 10.1371/journal.pone.0104770 (PMC4134228; doi:10.1371/journal.pone.0104770)
Supplement: Table S6 — MicroRNAs from Venn Diagram analysis. This table shows a list of microRNAs induced by WNV infection and polyI:C treatment in HEK293 cells. Each column details the microRNAs for the Venn diagram analysis (Figure 7). MicroRNAs unique to each treatment column are denoted in bold font with grey background. MicroRNAs that met the hard criteria defined for analysis in Figure 5 are denoted with a dash (−). (DOCX) [file pone.0104770.s008.docx]

**Table S6. MicroRNAs from Venn Diagram analysis: List of microRNAs induced by WNV infection and polyI:C treatment in HEK293 cells**

|  | | |
| --- | --- | --- |
| **HEK293-NULL + WNV** | **HEK293-TLR3+WNV** | **HEK293-TLR3 + pI:C** |
| 92 | 92 | 95 |
| 95 | 95 | 96 |
| 96 | 96 | **105** |
| 184 | 184 | **127** |
| 189 | 189 | **137** |
| 197 | 197 | **139** |
| 218 | 218 | **153** |
| 221 | 221 | **-186** |
| 301 | 301 | **192** |
| 331 | 331 | **205** |
| -367 | -367 | **216** |
| -372 | -372 | **219** |
| 382 | 382 | **220** |
| 410 | 410 | **325** |
| -412 | -412 | **328** |
| 432 | 432 | 331 |
| 433 | 433 | **335** |
| 483 | 483 | **368** |
| 486 | 486 | -372 |
| 491 | 491 | **373** |
| 492 | 492 | **375** |
| -496 | -496 | **379** |
| 500 | 500 | **381** |
| 503 | 503 | 382 |
| 508 | 508 | **-384** |
| 522 | 522 | **411** |
| 524 | 524 | -412 |
| 545 | 545 | **-429** |
| 549 | 549 | 432 |
| -561 | -561 | **455** |
| -562 | -562 | 483 |
| -563 | -563 | 486 |
| 571 | 571 | **488** |
| 572 | 572 | **490** |
| 573 | 573 | 491 |
| 575 | 575 | -496 |
| 584 | 584 | **499** |
| 585 | 585 | 508 |
| -588 | -588 | **521** |
| 614 | 614 | 522 |
| 615 | 615 | 524 |
| 636 | 636 | **555** |
| 641 | 641 | -561 |
| 643 | 643 | -562 |
| -651 | -651 | **564** |
| 653 | 653 | 571 |
| -655 | -655 | 572 |
| -656 | -656 | 573 |
| 659 | 659 | 575 |
| -662 | -662 | **-578** |
| 10a | 10a | **580** |
| 124a | 124a | 584 |
| -125b | -125b | 585 |
| 126# | 126# | **-586** |
| 133a | 133a | **599** |
| -155# | -155# | **603** |
| 18a | 18a | **-608** |
| 30a-5p | **302b** | **-613** |
| 369-3p | 30a-5p | 614 |
| 376a | 369-3p | 615 |
| -376a# | 376a | **616** |
| 380-5p | -376a# | 636 |
| 487b | 380-5p | 641 |
| 518c | 487b | 643 |
| 518f | 518c | 653 |
| 519a | 518f | **654** |
| -519d | **518f#** | 659 |
| 526c | 519a | **-661** |
| 548d | -519d | 662 |
| 7b | **519e** | **142-5p** |
|  | 526c | **146a** |
|  | **542-3p** | **148b** |
|  | 548d | -155# |
|  | 7b | **17-5p** |
|  |  | **182#** |
|  |  | **200a** |
|  |  | 302b |
|  |  | **34b** |
|  |  | 369-3p |
|  |  | 380-5p |
|  |  | **485-3p** |
|  |  | **485-5p** |
|  |  | 487b |
|  |  | **-493-3p** |
|  |  | **517a** |
|  |  | **517c** |
|  |  | 518c |
|  |  | 518f |
|  |  | 519a |
|  |  | -519d |
|  |  | 519e |
|  |  | **526a** |
|  |  | 548d |

List of microRNAs induced at 8 hours post-infection with WNV or post-treatment with polyI:C. Each column details the microRNAs for the Venn diagram analysis (Figure 7). MicroRNAs unique to each treatment column are denoted in bold font with grey background. MicroRNAs that met the hard criteria defined for analysis in Figure 5 are denoted with a dash (-).
