# Supplementary material for: Toll-Like Receptor-3 Is Dispensable for the Innate MicroRNA Response to West Nile Virus (WNV)
Source: PLoS One. 2014 Aug 15;9(8):e104770. doi: 10.1371/journal.pone.0104770 (PMC4134228; doi:10.1371/journal.pone.0104770)
Supplement: Table S7 — Ingenuity Functional Analysis of miRNA Targets from polyI:C induced microRNAs (Unique to pI:C treatment). MicroRNAs uniquely upregulated by polyI:C treatment in HEK293-TLR3 cells were analyzed using Ingenuity pathway analysis software. Gene ontology categories and functions of these microRNA targets are shown. (DOCX) [file pone.0104770.s009.docx]

**Table S7.**

**Ingenuity Functional Analysis of miRNA Targets from polyI:C induced microRNAs (Unique to pI:C treatment)**

| **GO Category** | **Function** | **p-Value** | **# Molecules** |
| --- | --- | --- | --- |
| Cellular Movement | Migration of cells | 1.15E-31 | 134 |
| Cellular Development | Differentiation of cells | 4.48E-31 | 132 |
| Cellular Growth and Proliferation | Proliferation of cells | 2.20E-30 | 199 |
| Cellular Movement | Invasion of cells | 4.30E-29 | 79 |
| Gene Expression | Transcription | 3.08E-26 | 114 |
| Cell Death and Survival | Apoptosis | 1.06E-24 | 164 |
| Cell Morphology | Morphology of cells | 4.83E-24 | 95 |
| DNA Replication | DNA synthesis | 1.10E-22 | 57 |
| Cell-To-Cell Signaling and Interaction | Activation of Cells | 1.12E-21 | 85 |
| Cellular Assembly and Organization | Cytoskeletal organization | 1.71E-18 | 80 |

MicroRNAs uniquely upregulated by polyI:C treatment in HEK293-TLR3 cells were analyzed using Ingenuity pathway analysis software. Gene ontology categories and functions of these microRNA targets are shown.
