# Supplementary material for: Toll-Like Receptor-3 Is Dispensable for the Innate MicroRNA Response to West Nile Virus (WNV)
Source: PLoS One. 2014 Aug 15;9(8):e104770. doi: 10.1371/journal.pone.0104770 (PMC4134228; doi:10.1371/journal.pone.0104770)
Supplement: Table S8 — Ingenuity Functional Analysis of miRNA Targets from abundantly expressed polyI:C induced microRNAs (Unique to pI:C treatment). Abundantly expressed microRNAs that were uniquely upregulated following polyI:C treatment in HEK293-TLR3 cells were identified using the hard cutoff criteria described for Figure 5. Targets of these microRNAs were analyzed using Ingenuity pathway analysis software and gene ontology categories and functions are shown. (DOCX) [file pone.0104770.s010.docx]

**Table S8.**

**Ingenuity Functional Analysis of miRNA Targets from abundantly expressed polyI:C induced microRNAs (Unique to pI:C treatment)**

| **GO Category** | **Function** | **p-Value** | **# Molecules** |
| --- | --- | --- | --- |
| Cellular Movement | Invasion of cells | 3.67E-14 | 38 |
| Cellular Assembly and Organization | Cytoplasmic organization | 2.61E-11 | 44 |
| Cellular Growth and Proliferation | Cell proliferation | 1.18E-08 | 48 |
| Cellular Assembly and Organization | Cell protrusion formation | 3.36E-08 | 27 |
| Molecular Transport | Protein transport | 1.04E-06 | 13 |
| Cell Cycle | Cell cycle progression | 1.46E-06 | 31 |
| Cellular Function and Maintenance | Formation of actin filaments | 1.53E-06 | 14 |

Abundantly expressed microRNAs that were uniquely upregulated following polyI:C treatment in HEK293-TLR3 cells were identified using the hard cutoff criteria described for Figure 5. Targets of these microRNAs were analyzed using Ingenuity pathway analysis software and gene ontology categories and functions are shown.
